# Acylcarnitine and Free Fatty Acid Profiles in Primary Biliary Cholangitis: Associations with Fibrosis and Inflammation

**DOI:** 10.3390/nu17071097

**Published:** 2025-03-21

**Authors:** Magdalena Rogalska, Agnieszka Błachnio-Zabielska, Piotr Zabielski, Jacek Robert Janica, Kamila Roszczyc-Owsiejczuk, Karolina Pogodzińska, Aleksandra Andrzejuk, Andrzej Dąbrowski, Robert Flisiak, Paweł Rogalski

**Affiliations:** 1 Department of Infectious Diseases and Hepatology, Medical University of Bialystok, 15-089 Białystok, Poland; robert.flisiak1@gmail.com; 2 Hygiene, Epidemiology and Metabolic Disorders Department, Medical University of Bialystok, 15-089 Białystok, Poland; agnieszka.blachnio-zabielska@umb.edu.pl (A.B.-Z.); kamila.roszczyc-owsiejczuk@umb.edu.pl (K.R.-O.); karolina.pogodzinska@umb.edu.pl (K.P.); 3 Department of Medical Biology, Medical University of Bialystok, 15-089 Białystok, Poland; piotr.zabielski@umb.edu.pl; 4 Department of Pediatric Radiology, Medical University of Bialystok, 15-089 Białystok, Poland; jrjanica@gmail.com; 5 Faculty of Medicine, Medical University of Bialystok, 15-089 Białystok, Poland; andrzejuk.aleksandra@gmail.com; 6 Department of Gastroenterology and Internal Medicine, Medical University of Bialystok, 15-089 Białystok, Poland; adabrows@umb.edu.pl (A.D.); pawel.rogalski@umb.edu.pl (P.R.)

**Keywords:** acylcarnitines, bile acids, fibrosis, free fatty acids, inflammation, liver stiffness, metabolic biomarkers, mitochondrial dysfunction, primary biliary cholangitis, β-oxidation

## Abstract

**Background:** Primary biliary cholangitis (PBC) is a chronic autoimmune liver disease characterized by bile duct destruction, cholestasis, and fibrosis. Acylcarnitines are esters of carnitine responsible for the transport of long-chain fatty acids into mitochondria for β-oxidation, playing a crucial role in energy metabolism and lipid homeostasis. This study aimed to assess acylcarnitine and free fatty acid (FFA) profiles in PBC patients and their associations with fibrosis severity and inflammation. **Methods:** This cross-sectional study included 46 PBC patients and 32 healthy controls. Acylcarnitines and FFAs were quantified using liquid chromatography-tandem mass spectrometry (LC-MS/MS) and enzymatic assays, respectively. Liver stiffness was measured by point shear wave elastography (ElastPQ), and fibrosis was assessed using APRI and FIB-4 scores. Inflammatory markers (IL-6, IL-1β) were also analyzed. **Results:** PBC patients had significantly higher levels of C18:1-acylcarnitine (median: 165.1 ng/mL) compared with the controls (152.4 ng/mL, *p* = 0.0036). Similarly, the FFA levels were markedly elevated in the PBC patients (median: 0.46 mM/L) compared with the controls (0.26 mM/L, *p* < 0.0001). Patients with higher liver stiffness (ElastPQ > 5.56 kPa) had significantly elevated C18:1-acylcarnitine (*p* = 0.0008) and FFA levels (*p* = 0.00098). Additionally, FFAs were significantly increased in patients with higher APRI and FIB-4 scores and were associated with elevated inflammatory markers (IL-6, IL-1β) and liver injury markers. Multivariate regression analysis confirmed C18:1-acylcarnitine (OR = 1.031, 95% CI: 1.007–1.057, *p* = 0.013) and FFAs (OR = 2.25 per 0.1 mM/L increase, 95% CI: 1.20–4.22, *p* = 0.012) as independent predictors of fibrosis severity in PBC. **Conclusions:** C18:1-acylcarnitine and FFAs are significantly elevated in PBC and are strongly associated with fibrosis severity and inflammation. These findings suggest a link between lipid metabolism disturbances and PBC. Both metabolites may potentially serve as non-invasive biomarkers of fibrosis progression in PBC, warranting further investigation.

## 1. Introduction

Primary biliary cholangitis (PBC) is a chronic autoimmune liver disease characterized by the progressive destruction of intrahepatic bile ducts. This process leads to cholestasis, chronic inflammation, and fibrosis, which in advanced stages can progress to cirrhosis and liver failure. PBC predominantly affects middle-aged women [1]. It is diagnosed based on elevated serum alkaline phosphatase (ALP) levels, the presence of anti-mitochondrial antibodies (AMA), and characteristic histological features [2]. At least two of these three criteria must be met for a definitive diagnosis. AMA-M2, detected in over 90% of patients, are highly specific autoantibodies that target mitochondrial enzymes, particularly the E2 component of the pyruvate dehydrogenase complex (PDC-E2), contributing to immune-mediated liver injury [3].

The liver, as a central organ in lipid metabolism, synthesizes bile acids from cholesterol, regulates lipoprotein metabolism, and facilitates fatty acid oxidation. In PBC, these processes are potentially altered due to cholestasis, chronic inflammation, and hepatocellular damage. Bile acid accumulation within hepatocytes leads to direct cytotoxic effects, oxidative stress, and apoptosis [4]. Cholestasis also disrupts the secretion of bile acids into the intestine, impairing lipid digestion and absorption, which further affects systemic lipid homeostasis. Disruption of the enterohepatic circulation of bile acids affects cholesterol metabolism, contributing to dyslipidemia, including decreased high-density lipoprotein (HDL) levels and the presence of lipoprotein X (Lp-X), an abnormal lipoprotein characteristic of cholestatic conditions [5].

Fatty acids transported to the liver are either utilized for energy production, incorporated into complex lipids, or converted into triglycerides for export as lipoproteins. Their mitochondrial β-oxidation depends on acylcarnitines, which facilitate the transport of long-chain fatty acids into mitochondria [6]. Alterations in acylcarnitine profiles have been linked to metabolic and mitochondrial disorders, but their role in PBC remains unclear. Free fatty acids (FFAs), released from triglycerides, play a dual role in cellular metabolism, serving as an energy source while also influencing inflammatory pathways. Changes in FFA metabolism have been observed in various liver diseases, but their significance in PBC-related metabolic dysfunction is not well-understood.

This study aimed to characterize acylcarnitine and FFA profiles in patients with PBC and assess their relationship with key clinical, biochemical, and imaging-based markers of disease severity. The accurate quantification of acylcarnitines and free fatty acids is essential for understanding their role in metabolic disturbances. In this study, acylcarnitines were measured using liquid chromatography-tandem mass spectrometry (LC-MS/MS) with an internal standard to ensure accuracy. The analysis involved sample extraction, centrifugation, and derivatization before chromatographic separation. Free fatty acids were quantified using an enzymatic assay with fluorometric measurements. Inflammatory markers were assessed using ELISA kits following standardized protocols. Specifically, the study examined associations between these metabolic parameters and inflammatory cytokines, non-invasive liver fibrosis indices, liver stiffness measurements obtained via point shear wave elastography, and portal hemodynamics assessed by Doppler ultrasound. Understanding these metabolic alterations may provide insights into the pathophysiology of PBC and help identify potential biomarkers for disease progression.

## 2. Methods

The study included 46 patients diagnosed with primary biliary cholangitis (PBC) and 32 healthy controls. It was conducted in accordance with the ethical principles outlined in the 1964 Declaration of Helsinki and its subsequent amendments. Written informed consent was obtained from all participants before their inclusion. The research protocol was reviewed and approved by the Bioethics Committee of our institution.

Patients were recruited during routine medical visits. All patients with PBC were treated with a standard dose (13–15 mg) of ursodeoxycholic acid (UDCA). Participants with conditions affecting liver function unrelated to PBC as well as those with severe systemic diseases or the use of medications that could influence lipid metabolism were excluded. The PBC group consisted predominantly of middle-aged women, which is consistent with the typical epidemiology of the disease. The control group was selected to match the PBC cohort in terms of age and sex distribution. Routine laboratory tests confirmed the absence of liver dysfunction or significant metabolic disorders in the control participants. Additionally, a detailed medical history was obtained for all participants to ensure comparability and minimize potential confounding factors. The control group mainly consisted of healthcare personnel.

### 2.1. Determination of Acylcarnitine Concentration

The concentration of acylcarnitine was determined using a modified version of the Giesbertz method [7]. Briefly, an internal standard (C17-carnitine) was added to each serum sample. The samples were then extracted using ice-cold methanol, centrifuged at 10,000× *g*/4 °C/10 min, and the resulting supernatant was dried under nitrogen into a fresh tube. The dried acylcarnitine extracts were subsequently derivatized to form butyl esters. This step involved shaking the samples at 60 °C for 20 min in 100 µL n-butanol containing 5% *v/v* acetyl chloride. The samples were then evaporated once more, reconstituted in 100 µL of methanol/water, and transferred to glass vials for UHPLC/MS/MS analysis. Quantification of acylcarnitines was performed on a Sciex QTRAP 6500+ triple quadrupole mass spectrometer (AB Sciex Germany GmbH, Darmstadt, Germany) using positive ion electrospray ionization (ESI) in multiple reaction monitoring (MRM) mode against standard curves prepared for each analyte. Chromatographic separation was carried out using ultra-performance liquid chromatography (Shimadzu Nexera-X2 UHPLC, Shimadzu Corporation, Kyoto, Japan). The analytical column used was a reversed-phase Zorbax SB-C18 column 2.1 × 150 mm, 1.8 µm (Agilent Technologies, Santa Clara, CA, USA).

### 2.2. Free Fatty Acid (FFA) Quantification

Free fatty acid levels were determined using the Free Fatty Acid Quantitation Kit (MAK044, Sigma-Aldrich, Saint Louis, MO, USA). The assay is based on a coupled enzymatic reaction, producing a colorimetric product measurable at 570 nm or a fluorometric signal (λ_ex = 535/λ_em = 590 nm), proportional to the fatty acid concentration. Briefly, serum samples and palmitic acid standards (1 nmol/µL) were prepared in a 96-well plate format, with standards ranging from 0 to 10 nmol/well for colorimetric detection. For sample preparation, 2 µL of Acyl-CoA Synthetase (ACS) reagent was added to each well, followed by incubation at 37 °C for 30 min to activate fatty acid conversion. After the initial incubation, 50 µL of the Master Reaction Mix (composed of Fatty Acid Assay Buffer, Fatty Acid Probe, Enzyme Mix, and Enhancer) was added to each well. The plate was gently mixed using a horizontal shaker and incubated again at 37 °C for 30 min, protected from light. Fluorescence intensity (λ_ex = 535/λ_em = 590 nm) was measured using a microplate reader. Quantification of FFAs was achieved by plotting a standard curve based on the palmitic acid standards, and the FFA concentrations in the samples were calculated accordingly.

### 2.3. Human IL-6 ELISA Assay

The concentration of interleukin-6 (IL-6) was measured using the Human IL-6 ELISA Kit (RAB0306, Sigma-Aldrich, Saint Louis, MO, USA). The assay is based on a sandwich ELISA method with a detection range of 1.37–1000 pg/mL and sensitivity below 3 pg/mL. Samples were diluted with the appropriate assay diluent, incubated with biotinylated detection antibody, and HRP-streptavidin. Absorbance was measured at 450 nm. The assay demonstrated recovery rates of 92–96% and CV values <10% (intra-assay) and <12% (inter-assay).

### 2.4. Human IL-1β ELISA Assay

Human IL-1β levels were measured using the Human IL-1β ELISA Kit (BMS224-2, Thermo Fisher Scientific, Vienna, Austria), which employs a sandwich ELISA format. Serum samples and standards (3.9–250 pg/mL) were added to pre-coated 96-well plates, followed by biotin-conjugated antibodies, streptavidin-HRP, and a TMB substrate. After a 10-min incubation, the reaction was stopped with phosphoric acid, and the absorbance was read at 450 nm. Concentrations were determined using a standard curve and adjusted for a 1:2 dilution factor. The assay’s detection limit is 0.3 pg/mL, ensuring high sensitivity for research applications.

### 2.5. Liver Stiffness and Doppler Ultrasound Assessment

Liver stiffness was assessed using point quantification elastography (ElastPQ) with a Philips ultrasound system. Due to the lack of official standards for liver stiffness assessed using ElastPQ in PBC, we adopted a threshold of 5.56 kPa as the value for significant fibrosis. This cutoff was based on the study by Mare et al., which included patients with PBC and established this value as a reference for fibrosis assessment using ElastPQ [8]. Measurements were performed by an experienced radiologist, with ten valid measurements obtained per patient. Median liver stiffness values were recorded in kilopascals (kPa), and results with an interquartile range (IQR) exceeding 30% of the median were excluded to ensure accuracy.

Doppler ultrasound was used to evaluate the portal circulation parameters including spleen size, portal vein diameter, splenic vein diameter, and hemodynamic indices of portal perfusion. All assessments were conducted by a single expert radiologist, ensuring consistency and minimizing variability.

### 2.6. Statistical Analysis

The statistical analysis was performed using Data Science Workbench, version 14 (Cloud Software Group, Inc., Fort Lauderdale, FL, USA, 2023). Data normality was assessed with the Shapiro–Wilk and Lilliefors tests, and variance homogeneity with Levene’s and Brown–Forsythe’s tests. Depending on these results, group comparisons were conducted using the Student’s *t*-test, Welch’s *t*-test, or the Mann–Whitney U test. Correlations were analyzed using Spearman’s rank coefficient. Multivariate logistic regression identified independent predictors of liver fibrosis, with odds ratios (ORs) and 95% confidence intervals (CIs) reported. Receiver operating characteristic (ROC) curves and Youden’s index determined the optimal cutoffs for fibrosis markers. Statistical significance was set at *p* < 0.05.

## 3. Results

The age and gender distribution did not differ significantly between the groups (Control: 58.8 ± 5.6 years, 96.9% female, n = 32; PBC: 61.8 ± 7.6 years, 95.7% female, n = 46; *p* = 0.0593 for age, *p* = 0.7824 for gender). Table 1 summarizes the basic laboratory parameters and non-invasive markers of liver fibrosis in the healthy controls and patients with primary biliary cholangitis (PBC). In patients with PBC, the platelet count was slightly lower, and the liver enzyme levels (including AST and ALT) were modestly elevated compared to controls. Additionally, inflammatory markers such as CRP and IL-6 were somewhat higher in the PBC group, although these values remained near normal. These statistically significant differences suggest that PBC patients experienced mild liver injury and a modest inflammatory response, which may indicate the early stages of liver fibrosis (Table 1).

**Figure 1:**
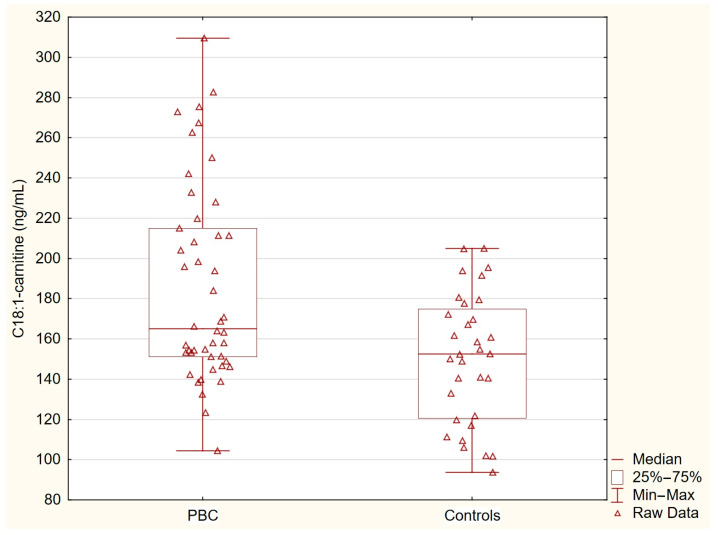
C18:1-carnitine in PBC group and control group. C18:1-carnitine concentrations were significantly elevated in patients with PBC compared with the healthy controls (*p* = 0.0036).

**Figure 2:**
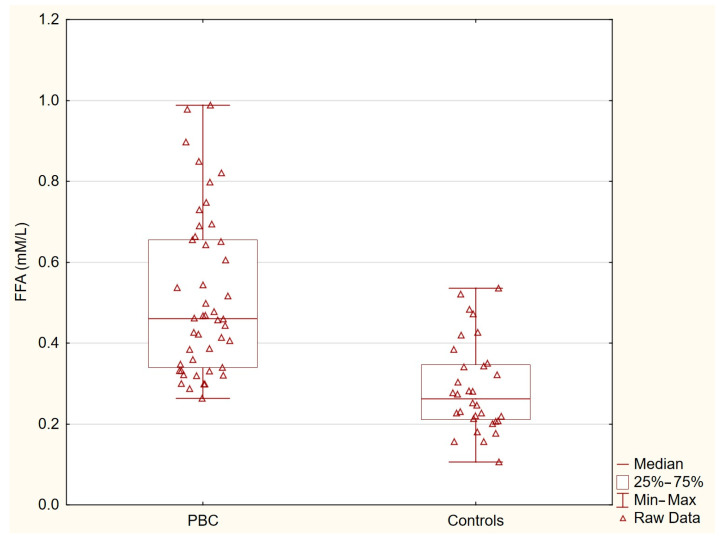
Free fatty acids in PBC group and control group. Free fatty acid levels were markedly elevated in patients with PBC compared with the healthy controls (*p* < 0.0001).

Table 2 presents the concentrations of acylcarnitines and free fatty acids in the healthy controls and patients with primary biliary cholangitis (PBC). The levels of C14-carnitine, C16-carnitine, and C18-carnitine were similar between the two groups. In contrast, the concentration of C18:1-carnitine was significantly higher in the PBC group (*p* = 0.0036), and the free fatty acid levels were markedly elevated in the PBC patients (*p* < 0.0001). These statistically significant differences indicate specific alterations in lipid metabolism associated with PBC (Table 2, Figure 1 and Figure 2).

**Figure 3:**
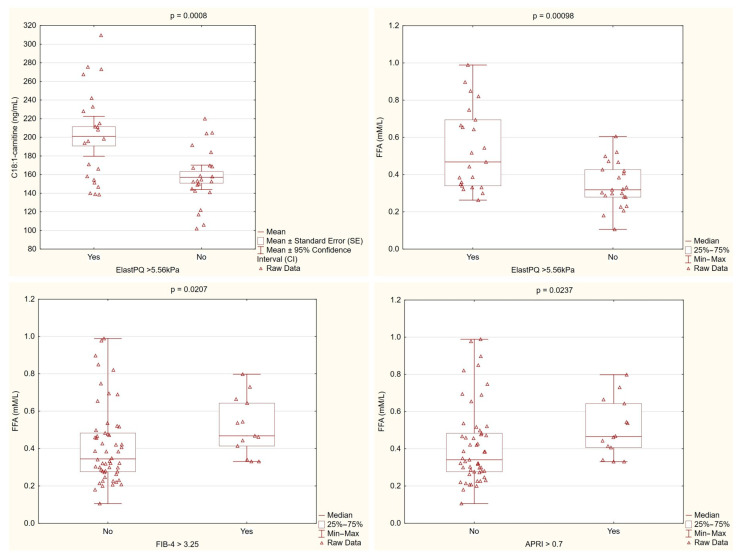
Lipid metabolism parameters stratified by fibrosis markers. C18:1-carnitine levels were significantly higher in patients with ElastPQ > 5.56 kPa compared with those with lower ElastPQ values (*p* = 0.0008). Similarly, the free fatty acid (FFA) levels were markedly elevated in patients with higher ElastPQ measurements (*p* = 0.00098) and differed significantly when stratified by the FIB-4 (*p* = 0.0207) and APRI (*p* = 0.0237) cutoffs. These findings support an association between altered lipid metabolism and increased liver stiffness/fibrosis in PBC.

Patients with higher liver stiffness, as measured by ElastPQ, consistently showed elevated levels of C18:1-carnitine and free fatty acids. These findings, along with differences observed in the non-invasive fibrosis markers, suggest that dysregulated lipid metabolism is linked to increased liver fibrosis in PBC (Figure 3).

Correlation analyses revealed that the C18:1-carnitine levels were primarily associated with fibrosis-related parameters, showing significant positive correlations with non-invasive fibrosis markers (APRI, FIB-4) and liver stiffness measured by ElastPQ. In contrast, the FFA levels were linked not only to fibrosis markers (APRI, FIB-4, ElastPQ), but also to inflammatory markers (IL-6, IL-1β) and liver enzymes reflecting liver injury (AST, ALT) and cholestasis (GGTP, ALP), see Table 3.

Doppler ultrasound assessments of portal circulation revealed significant correlations between the lipid parameters and hemodynamic markers in PBC. The C18:1-carnitine levels were positively associated with the spleen length (r = 0.49, *p* < 0.001), spleen cross-sectional area (r = 0.47, *p* < 0.001), and portal vein dimensions. Similarly, the FFA levels showed significant correlations with multiple parameters of portal circulation including the superior mesenteric vein diameter (r = 0.37, *p* = 0.0072), splenic perfusion index (r = 0.29, *p* = 0.0371), and splenic vein measurements. These findings suggest that alterations in lipid metabolism, particularly elevated C18:1-carnitine and FFA levels, are associated with disturbances in portal hemodynamics and the presence of portal hypertension in PBC (Table 4).

### Multivariate Logistic Regression Analysis of Fibrosis Predictors in PBC

The analysis aimed to identify independent predictors of significant liver fibrosis (ElastPQ > 5.56 kPa) in primary biliary cholangitis (PBC) patients (8). The following quantitative predictors were initially considered: APRI, FIB-4, platelet count (PLT), GGTP, spleen length, IL-6, C18:1-carnitine (ng/mL), and FFA (mM/L). GGTP and IL-6 were excluded from further modeling due to non-significance in univariate analysis (*p* > 0.05). Two separate multivariate logistic regression models were developed: one including FIB-4 and C18:1-carnitine, and the other including spleen length and FFA. Both models demonstrated strong discriminative ability and good model fit.

In the C18:1-carnitine model, each 1 ng/mL increase in C18:1-carnitine was associated with a 3.1% increase in fibrosis risk (OR = 1.031, 95% CI: 1.007–1.057, *p* = 0.013), while FIB-4 remained a strong predictor (OR = 2.94, 95% CI: 1.19–7.29, *p* = 0.020). The model demonstrated good overall fit, with a Nagelkerke R^2^ of 0.558, and the Hosmer–Lemeshow test (*p* = 0.483) indicated no significant lack of fit, confirming adequate calibration (Table 5).

In the FFA model, both FFA and spleen length were significant predictors of fibrosis. Each 0.1 mM/L increase in FFA was associated with a 2.25-fold increase in fibrosis risk (OR = 2.25, 95% CI: 1.20–4.22, *p* = 0.012), while the spleen length was also predictive (OR = 2.40, 95% CI: 1.21–4.75, *p* = 0.012). The model showed strong performance, with a Nagelkerke R^2^ of 0.619 and a Hosmer–Lemeshow test *p*-value of 0.298, confirming good calibration and reliability in fibrosis prediction (Table 6).

Both models confirmed that altered lipid metabolism is strongly associated with liver fibrosis in PBC. While C18:1-carnitine exhibited higher specificity, FFA had greater sensitivity, suggesting that both markers provide complementary insights into fibrosis progression. The predictive performance remained strong in cross-validation, supporting the potential use of C18:1-carnitine, FFA, and FIB-4 as non-invasive biomarkers for fibrosis risk stratification in PBC patients.

**Figure 4:**
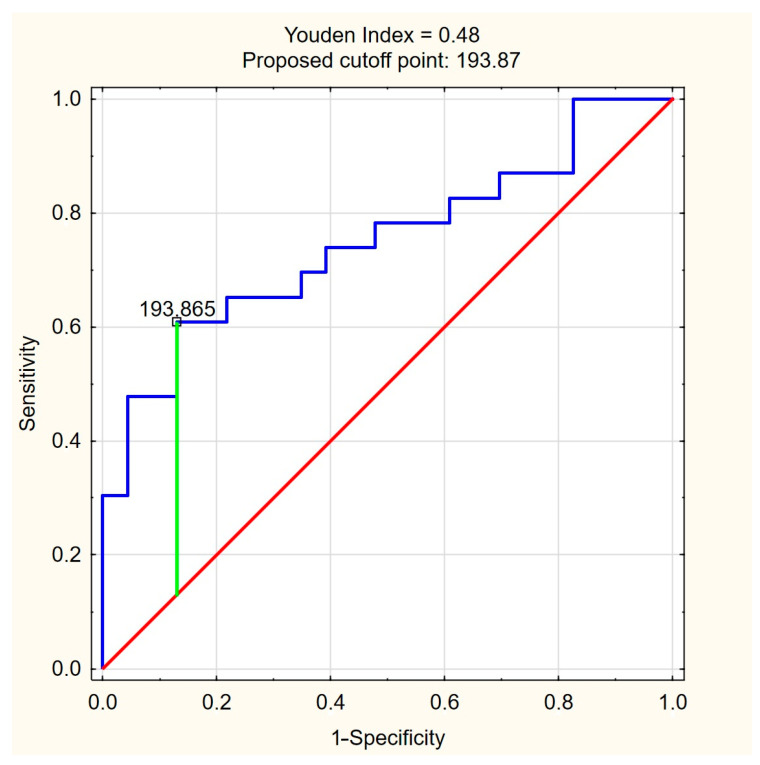
ROC curve for C18:1-carnitine as a predictor of significant liver fibrosis (ElastPQ > 5.56 kPa) in PBC. The optimal cutoff of 193.87 ng/mL was determined based on the highest Youden index (0.48), maximizing specificity (87.0%) while maintaining moderate sensitivity (60.9%). Blue line—ROC curve, red line—baseline, green line—optimal cutoff (Youden index).

**Figure 5:**
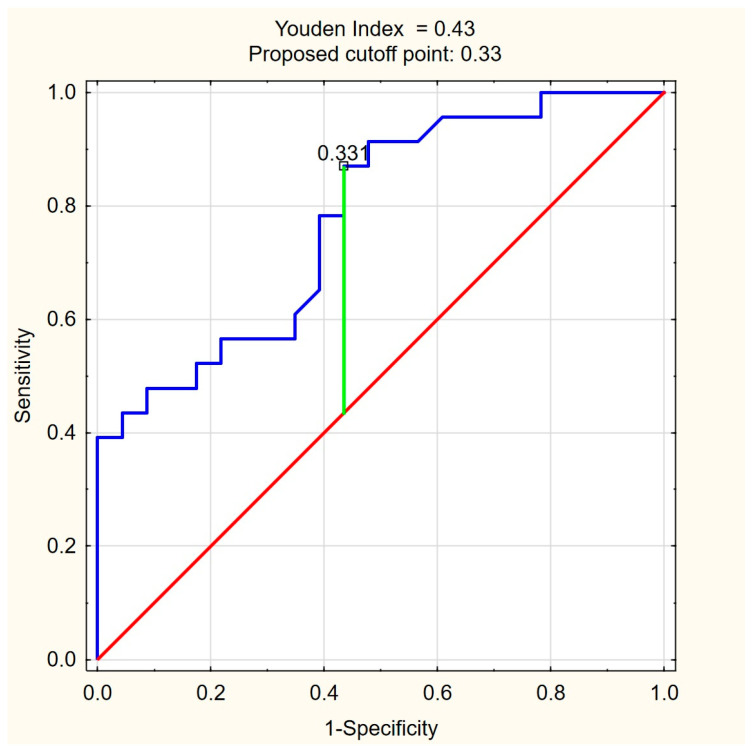
ROC curve for FFA (mM/L) as a predictor of significant liver fibrosis (ElastPQ > 5.56 kPa) in PBC. The optimal cutoff of 0.33 mM/L was determined based on the highest Youden index (0.43), balancing high sensitivity (87.0%) with moderate specificity (56.5%). Blue line—ROC curve, red line—baseline, green line—optimal cutoff (Youden index).

The ROC curves for C18:1-carnitine and FFAs (Figure 4 and Figure 5) illustrate the discriminative ability of both models. The optimal cutoff for C18:1-carnitine was determined as 193.87 ng/mL, based on the highest Youden index (0.48), maximizing specificity (87.0%) while maintaining moderate sensitivity (60.9%). For FFAs, the optimal cutoff was 0.33 mM/L, corresponding to a Youden index of 0.43, balancing high sensitivity (87.0%) with moderate specificity (56.5%). These results indicate that C18:1-carnitine is a high-specificity marker, whereas FFA is a high-sensitivity marker, supporting their complementary roles as non-invasive fibrosis biomarkers in PBC.

## 4. Discussion

This study provides novel insights into alterations in lipid metabolism in patients with primary biliary cholangitis (PBC). Using targeted liquid chromatography-tandem mass spectrometry (LC-MS/MS), we quantified the acylcarnitine species, while enzymatic assays were used to measure free fatty acid (FFA) levels in patients with PBC and the healthy controls. Additionally, non-invasive liver fibrosis markers and liver stiffness measurements obtained via shear wave elastography were assessed to explore potential associations between metabolic alterations and fibrosis progression. Our findings demonstrate that patients with PBC have significantly elevated levels of C18:1-carnitine and FFAs compared with healthy controls. To our knowledge, this is the first study to comprehensively evaluate long-chain acylcarnitine and FFA profiles in patients with PBC and explore their potential role as metabolic biomarkers of disease severity.

The primary physiological function of acylcarnitines is to transport fatty acids from the cytosol into the mitochondrial matrix, where they undergo β-oxidation to produce energy. They are classified by chain length as short-chain (C2–C5), medium-chain (C6–C12), long-chain (C13–C20), and very long-chain (>C21) and further divided into saturated and unsaturated forms. While short- and medium-chain acylcarnitines can diffuse freely across mitochondrial membranes, long-chain acylcarnitines require the carnitine shuttle for mitochondrial entry. This transport system involves carnitine palmitoyltransferase 1 (CPT1), which catalyzes the conversion of fatty acyl-CoA into acylcarnitines on the outer mitochondrial membrane, carnitine-acylcarnitine translocase (CACT), which facilitates their transfer across the inner mitochondrial membrane, and carnitine palmitoyltransferase 2 (CPT2), which reconverts acylcarnitines into acyl-CoA inside the mitochondrial matrix, enabling β-oxidation [9,10].

Carnitine homeostasis is regulated by biosynthesis, dietary intake, and renal reabsorption via the organic cation transporter OCTN2 (novel organic cation transporter 2). The heart, skeletal muscle, and liver are the primary sources of circulating acylcarnitines, reflecting their key role in fatty acid oxidation. C18:1-acylcarnitine, also known as oleoylcarnitine, is a long-chain acylcarnitine derived from monounsaturated oleic acid, conjugated with L-carnitine. Unlike fully saturated acylcarnitines, its structure contains a double bond, which necessitates an additional enzymatic step in β-oxidation. This step involves enoyl-CoA isomerase, which converts the cis-double bond into a trans configuration, allowing further processing within the mitochondrial fatty acid oxidation pathway. This structural characteristic may influence its metabolism and function in fatty acid oxidation [6,11].

The diagnostic potential of acylcarnitines has been well-established in inborn metabolic disorders, where their altered profiles serve as biomarkers of fatty acid oxidation defects. In recent years, their role has expanded beyond metabolic diseases to include diabetes, cardiovascular diseases, and inflammatory conditions. Long-chain acylcarnitines, apart from their function in oxidative catabolism, have been implicated in cardiac electrophysiology, insulin signaling, cellular stress, and inflammation. Some evidence suggests that acylcarnitines could serve as diagnostic biomarkers for hepatocellular carcinoma (HCC), but further research is required to determine their clinical significance [9,12,13,14,15].

In this study, we observed that the C18:1-acylcarnitine levels were significantly higher in PBC patients compared with the healthy controls. This increase was consistently associated with markers of liver fibrosis including liver stiffness, APRI, and FIB-4 as well as portal circulation abnormalities. Further analysis demonstrated that the C18:1-acylcarnitine levels were significantly higher in patients with ElastPQ > 5.56 kPa, and in multivariate logistic regression, C18:1-acylcarnitine remained an independent predictor of liver fibrosis in PBC, reinforcing its potential diagnostic significance. These findings suggest that C18:1-acylcarnitine may be linked to fibrosis severity in PBC, though the underlying mechanisms remain unclear. While further research is needed, increased C18:1-acylcarnitine levels could serve as a biomarker for fibrosis severity in PBC, providing potential diagnostic insight.

Despite the liver being one of the primary organs involved in acylcarnitine metabolism, its role in chronic liver diseases remains poorly understood. A study by Miyaaki et al. reported increased C16- and C18:1-acylcarnitine levels in patients with liver cirrhosis of different etiologies, with a significant correlation between these levels and the severity of liver dysfunction, as indicated by the Child–Pugh score. Notably, the C18:1 levels were consistently high in patients with a Child–Pugh score >11, regardless of the underlying cause of cirrhosis [16]. In contrast, our study focused on PBC patients at an early disease stage. Despite the absence of advanced liver dysfunction, we observed a significant elevation of C18:1-acylcarnitine, which correlated with markers of liver fibrosis. This suggests that C18:1-acylcarnitine may be associated with fibrosis progression in PBC, independent of cirrhosis.

In this study, we also observed that the FFA levels were significantly higher in the PBC patients compared with the healthy controls. This increase was strongly associated with markers of liver fibrosis including liver stiffness and non-invasive fibrosis indices (APRI, FIB-4). Additionally, elevated FFA levels correlated with inflammatory cytokines (IL-6, IL-1β) and markers of hepatocellular injury, suggesting a link between FFA metabolism, inflammation, and liver damage in PBC. Further analysis revealed that the FFA levels were elevated in patients with greater liver stiffness, indicating that disturbances in FFA metabolism may accompany fibrotic changes in PBC. In multivariate logistic regression, FFAs remained an independent predictor of liver fibrosis. While the exact mechanisms linking FFAs to fibrosis progression remain unclear, their role in hepatic inflammation and oxidative stress suggests that they may contribute to fibrogenesis in PBC. Previous studies suggest that free fatty acids can modulate inflammatory pathways, and the release of pro-inflammatory cytokines such as TNF-α and IL-6. This mechanism may contribute to hepatic inflammation and disease progression in cholestatic liver diseases. These findings support the hypothesis that FFA accumulation in PBC is linked to both metabolic and inflammatory disturbances, potentially influencing the progression of liver damage resulting in liver fibrosis and cirrhosis [17,18,19,20]. The interplay between acylcarnitines, FFAs, and liver fibrosis in PBC highlights the metabolic–immune–inflammatory axis as a critical driver of disease progression. While FFAs are directly involved in inflammatory signaling and oxidative stress, acylcarnitines may serve as markers of mitochondrial dysfunction and altered lipid metabolism, indirectly contributing to the fibrotic process. Further research is needed to determine whether these metabolic alterations actively influence fibrosis progression or are secondary to ongoing liver injury.

Elevated free fatty acid (FFA) levels observed in PBC patients may indicate disturbances in fatty acid metabolism including impaired β-oxidation. Several mechanisms may contribute to this dysfunction in cholestatic liver diseases.

One potential cause is mitochondrial toxicity induced by bile acid accumulation, which can reduce the activity of β-oxidation enzymes, disrupt the electron transport chain (ETC), decrease ATP production, and increase oxidative stress, ultimately leading to mitochondrial damage. Bile acid accumulation not only exerts cytotoxic effects, but also interferes with lipid metabolism by modulating nuclear receptors, particularly FXR (farnesoid X receptor) and PPARα (peroxisome proliferator-activated receptor alpha). Under physiological conditions, FXR helps regulate the balance between fatty acid oxidation and lipogenesis. However, in cholestasis, reduced FXR activation leads to the upregulation of lipogenic genes and enhanced de novo fatty acid synthesis, while mitochondrial dysfunction further impairs β-oxidation, promoting FFA accumulation [21,22].

Another possible factor is the accumulation of long-chain acylcarnitines, which has been linked to mitochondrial toxicity, inflammation, and oxidative stress. An excess of acylcarnitines may result from a bottleneck in β-oxidation, further exacerbating metabolic imbalances. The elevated C18:1 levels observed in our study may reflect a blockade in β-oxidation and mitochondrial dysfunction. However, acylcarnitines have also been proposed to serve a protective role by facilitating the detoxification of excess lipids and mitigating bile acid toxicity. This suggests that acylcarnitine accumulation in PBC may be a consequence of disrupted β-oxidation but could also represent an adaptive metabolic response [6,10].

Additionally, cholestasis impairs the normal flow of bile acids into the intestine, disrupting lipid digestion and absorption. Since bile acids facilitate the emulsification and breakdown of dietary fats, their deficiency leads to the malabsorption of lipids including fatty acids derived from triglyceride hydrolysis. As a compensatory response, hepatic lipogenesis is upregulated, contributing to increased circulating FFA levels [21,23].

Among the cholestatic liver diseases, primary biliary cholangitis (PBC) is uniquely associated with a potential predisposition to mitochondrial dysfunction due to the presence of anti-mitochondrial antibodies (AMA-M2). These autoantibodies, detected in over 90% of PBC patients, specifically target the E2 component of the pyruvate dehydrogenase complex (PDC-E2), which is located on the inner mitochondrial membrane [2,24]. The PDC is a key enzyme complex that catalyzes the conversion of pyruvate to acetyl-CoA, linking glycolysis with the tricarboxylic acid (TCA) cycle, and plays a central role in cellular energy metabolism. Although mitochondrial dysfunction has not been directly studied in the context of AMA-M2-related metabolic alterations, it remains a plausible factor contributing to metabolic disturbances in PBC. Given that PDC-derived acetyl-CoA fuels the TCA cycle, impaired PDC function may force cells to rely more heavily on fatty acid oxidation as an alternative source of acetyl-CoA for energy production. This shift could influence acylcarnitine and FFA metabolism, further affecting mitochondrial homeostasis. Since acylcarnitines mediate the transport of fatty acids into mitochondria for β-oxidation, and FFAs undergo β-oxidation to generate acetyl-CoA for the TCA cycle, disruptions in PDC activity, fatty acid oxidation, and acylcarnitine metabolism may be interconnected in PBC. While this remains a theoretical concept, further research is needed to determine whether such metabolic interactions contribute to disease pathology.

Our findings suggest that metabolic disturbances in PBC, particularly altered FFA metabolism and acylcarnitine accumulation, could be potential targets for future dietary or pharmacological interventions. Addressing these metabolic imbalances through personalized nutrition strategies or novel therapies aimed at modulating fatty acid oxidation and bile acid metabolism may help slow fibrosis progression and improve clinical outcomes. It should be noted that all patients in our study group were treated with a standard dose (13–15 mg/kg) of ursodeoxycholic acid (UDCA), which is known for its potential to induce a biochemical response in PBC. Standard UDCA treatment helps reduce inflammation, inhibit apoptosis, lower blood bile acid concentrations, and improve fat digestion. However, its effects on disease progression are limited, particularly regarding lipid metabolism and fibrosis [25]. Obeticholic acid (OCA), a second-line drug for PBC, has an unfavorable impact on the lipid profile in patients with PBC, as it has been associated with a reduction in HDL and an increase in LDL [26]. Most concerns regarding hypolipidemic drugs stem from their potential hepatotoxicity; however, both statins and fibrates appear to be effective and safe options for patients with PBC. While both drug classes can reduce the free fatty acid (FFA) concentrations, the effect is more pronounced with fibrates [27,28].

A structured nutrition plan is also essential for managing patients with PBC. Preventing prolonged fasting through regular carbohydrate intake helps mitigate acylcarnitine accumulation. The nutritional strategy for fatty acid oxidation disorders emphasizes a targeted macronutrient composition, including limiting the consumption of long-chain fatty acids (such as oleic acid) and incorporating medium-chain triglycerides (MCTs) (e.g., coconut oil) to facilitate rapid absorption, thereby bypassing hepatic metabolism and optimizing energy utilization [29]. Individualized dietary counseling by a professional nutritionist should always be obtained and integrated into comprehensive disease management. The presence of trained nutritionists, particularly in tertiary care hospitals, is essential to ensure the implementation of evidence-based dietary strategies, as highlighted in recent studies emphasizing the role of clinical nutrition in chronic disease management [30].

Despite its novel findings, this study had certain limitations. First, the sample size was relatively small, which may limit the generalizability of our results. Larger, multicenter studies are needed to validate our findings in a broader PBC population. Second, while our study identified significant associations between lipid metabolism alterations and fibrosis markers, its cross-sectional design did not allow us to determine the causal relationships. Additionally, our study did not directly assess mitochondrial function, leaving the link between AMA-M2-related metabolic disturbances and β-oxidation impairment unverified. Further research, including in vitro and in vivo models of cholestatic liver disease, could help determine whether disturbances in lipid metabolism contribute to fibrosis progression or are a consequence of the disease.

A key aspect of our study was the observed increase in long-chain acylcarnitines, which suggests disturbances in mitochondrial β-oxidation. Although we did not directly assess mitochondrial function, this conclusion is based on well-established metabolic principles. Acylcarnitines are formed in both peroxisomes and mitochondria, but long-chain acylcarnitines (>C14) are produced exclusively within mitochondria, making them reliable indicators of β-oxidation efficiency. The accumulation of these metabolites is widely recognized in the diagnosis of inherited disorders of fatty acid oxidation and mitochondrial dysfunction. Therefore, while further research is needed to directly evaluate mitochondrial activity in PBC, our findings strongly suggest an association between impaired β-oxidation and lipid metabolism disturbances in this disease.

## 5. Conclusions

This study provides new insights into lipid metabolism alterations in PBC, demonstrating that C18:1-acylcarnitine and FFAs are significantly elevated in PBC patients and correlate with fibrosis severity and inflammatory markers. Our findings suggest that disturbances in β-oxidation and lipid metabolism may play a role in PBC pathophysiology. While the exact mechanisms remain unclear, the interplay between acylcarnitines, FFAs, and liver fibrosis in PBC highlights the metabolic–immune–inflammatory axis as a critical driver of disease progression, and these metabolic alterations could serve as potential non-invasive biomarkers of disease severity. Further research is needed to explore their clinical utility and determine whether targeting metabolic pathways could offer new therapeutic strategies for PBC.

## Figures and Tables

**Table 1:** The results of basic laboratory tests and non-invasive liver fibrosis markers.

	Controls (n = 32)	PBC Group (n = 46)	*p*
WBC (×10^3^/µL)	6.4 ± 1.8	5.7 ± 2.1	0.1864
HGB (g/dL)	13.7 (12.9; 14.2)	12.95 (12.1; 14)	0.0539
PLT (×10^3^/µL)	247.6 ± 63.7	184.7 ± 81.4	0.0011
PT (sec.)	13.4 (12.7; 13.8)	12.6 (11.9; 13.1)	0.0088
Fibrinogen (mg/dL)	344.2 ± 53.2	401.7 ± 75.7	0.0014
Bilirubin (mg/dL)	0.6 (0.5; 0.7)	0.8 (0.6; 1.4)	0.0118
Creatinine (mg/dL)	0.7 (0.6; 0.8)	0.8 (0.7; 0.9)	0.18
CRP (mg/L)	1.2 (1; 3)	4.65 (2.2; 7.5)	<0.0001
IL-6 (pg/mL)	234.5 (158.2; 300.7)	352.9 (252; 573)	0.0002
AST (IU/L)	19 (16; 24)	33 (26; 43)	<0.0001
ALT (IU/L)	17 (15; 21)	28 (22; 45)	<0.0001
GGTP (IU/L)	20 (17; 24)	68 (43; 96)	<0.0001
ALP (IU/L)	62 (56; 68)	124 (93; 173)	<0.0001
Albumin (g/dL)	3.98 ± 0.28	3.78 ± 0.42	0.4874
Ammonia (µmol/L)	31.7 (23.8; 34.6)	36.2 (30; 47.8)	0.0888
APRI	0.22 (0.17; 0.24)	0.45 (0.27; 0.87)	<0.0001
FIB-4	1.2 (0.9; 1.3)	1.8 (1.4; 3.6)	<0.0001
ElastPQ (kPa)	3.3 (2.9; 3.9)	6.7 (5.2; 8.3)	<0.0001

Abbreviations: Alanine aminotransferase (ALT), alkaline phosphatase (ALP), APRI (AST to platelet ratio index), aspartate aminotransferase (AST), C-reactive protein (CRP), point quantification elastography (ElastPQ), fibrosis-4 index (FIB-4), gamma-glutamyl transpeptidase (GGTP), hemoglobin (HGB), interleukin-6 (IL-6), platelet count (PLT), primary biliary cholangitis (PBC), prothrombin time (PT), white blood cell count (WBC).

**Table 2:** The results of the acylcarnitine and free fatty acid concentrations.

	Controls (n = 32)	PBC Group (n = 46)	*p*
C14-carnitine (ng/mL)	12.9 (11.5; 18)	13.8 (11.3; 17.9)	0.8046
C16-carnitine (ng/mL)	40.5 (36.5; 49.1)	39.1 (35.1; 45.9)	0.4699
C18:1-carnitine (ng/mL)	152.4 (120.7; 174.9)	165.1 (151.2; 214.8)	0.0036
C18-carnitine (ng/mL)	21.6 ± 5.8	20.2 ± 5.9	0.3128
FFA (mM/L)	0.26 (0.21; 0.35)	0.46 (0.34; 0.65)	<0.0001

Abbreviations: FFAs (free fatty acids), PBC (primary biliary cholangitis).

**Table 3:** Correlations of C18:1-carnitine and free fatty acids with fibrosis, inflammation, and liver injury markers in PBC.

Lipid Marker	Parameter	Spearman R	*p*-Value
C18:1-carnitine	APRI	0.44	0.00018
C18:1-carnitine	FIB-4	0.46	0.00008
C18:1-carnitine	ElastPQ (kPa)	0.66	<0.0001
C18:1-carnitine	Platelet Count	−0.29	0.0132
FFA	APRI	0.45	0.00011
FFA	FIB-4	0.35	0.00323
FFA	ElastPQ (kPa)	0.37	0.01141
FFA	AST	0.49	0.00002
FFA	ALT	0.53	0.000003
FFA	GGTP	0.34	0.01422
FFA	ALP	0.41	0.00248
FFA	IL-6	0.43	0.00010
FFA	IL-1β	0.26	0.02142

Abbreviations: Aspartate aminotransferase (AST), alanine aminotransferase (ALT), alkaline phosphatase (ALP), AST to platelet ratio index (APRI), point quantification elastography (ElastPQ), fibrosis-4 index (FIB-4), free fatty acids (FFAs), gamma-glutamyl transferase (GGTP), interleukin-6 (IL-6), interleukin-1 beta (IL-1β).

**Table 4:** Correlations of C18:1-carnitine and free fatty acids with spleen and portal hemodynamic parameters in PBC.

Lipid Marker	Parameter	Spearman R	*p*-Value
C18:1-carnitine	Spleen Length	0.49	0.00014
C18:1-carnitine	Spleen Cross-Sectional Area	0.47	0.00026
C18:1-carnitine	Splenic Index	0.41	0.00167
C18:1-carnitine	Portal Vein Diameter	0.28	0.04239
C18:1-carnitine	Portal Vein Area	0.33	0.01678
FFA	Spleen Length	0.28	0.04164
FFA	Spleen Cross-Sectional Area	0.27	0.04474
FFA	Splenic Perfusion Index	0.29	0.03705
FFA	Hepatic Artery TAM	0.38	0.00652
FFA	Hepatic Vein DI	0.37	0.00878
FFA	Superior Mesenteric Vein Diameter	0.37	0.00720
FFA	Splenic Vein Diameter	0.41	0.00228
FFA	Splenic Vein Area	0.41	0.00257
FFA	Splenic Vein Volume Flow	0.39	0.00428

Abbreviations: free fatty acids (FFAs), time-averaged mean velocity (TAM), diastolic index (DI).

**Table 5:** Predictors of significant liver fibrosis (ElastPQ > 5.56 kPa) in the C18:1-carnitine model.

Characteristic	Coefficient (β)	SE	*p*-Value	OR	95% CI for OR
Intercept	–7.64	2.49	0.0022	—	—
C18:1-Carnitine (ng/mL)	0.03094	0.01247	0.0131	1.031	1.007–1.057
FIB-4	1.08	0.46	0.0196	2.94	1.19–7.29

**Table 6:** Predictors of significant liver fibrosis (ElastPQ > 5.56 kPa) in the FFA model.

Characteristic	Coefficient (β)	SE	*p*-Value	OR	95% CI for OR
Intercept	–12.134	4.016	0.003	—	—
FFA (per 0.1 mM/L)	0.8104	0.3216	0.012	2.25	1.20–4.22
Spleen Length	0.875	0.348	0.012	2.40	1.21–4.75

## Data Availability

The raw data supporting the conclusions of this article will be made available by the authors on request.
